# Editorial Prudence

**Published:** 1915-08

**Authors:** 


### Editorial Prudence.—Medical Economics.

One of our contemporaries expresses the belief that if it were not for the demand for hygienists and sanitarians, there would be no room for physicians, as their occupation—that of treating diseases—will be practically gone. The slump in medical students would cause 100 colleges to close. For some months, we have been at work collecting statistics as to the number of official positions open to physicians and paying living salaries. It has been surprisingly difficult to secure such statistics. At the risk of being imprudent ourself, we may say that the total number of physicians employed by all branches of the government on living salaries is probably about sufficient to give employment to the graduates of one year. About 2% of such positions would naturally become vacant annually. There are probably about as many more semi-official positions available. Last year, we counted a total of 101 medical colleges in the U. S., giving degrees. Now, granting that there are too many physicians, and that prophylaxis and hygiene have diminished disease, quite a number of our acquaintances admit that they are still fairly busy with practice. There is no immediate prospect of the elimination of disease and accidents or of reproduction. Physicians will be needed in moderate numbers for at least another decade and even the hygienic and sanitary work mentioned will give places for the graduates of four average schools. We think, therefore, that not more than 97 medical schools will have to close their doors in the near future.

